# Cognitive Reactivity: Cultural Adaptation and Psychometric Testing of the Persian Version of the Leiden Index of Depression Sensitivity Revised (LEIDS-R) in an Iranian Sample

**DOI:** 10.1007/s11469-016-9713-z

**Published:** 2016-11-14

**Authors:** Shahla Ostovar, Mariani Binti Md Nor, Mark D. Griffiths, Soghra Akbari Chermahini

**Affiliations:** 1 Department of Educational Psychology and Counselling, Faculty of Education, University of Malaya, 50603 Kuala Lumpur, Malaysia; 2 Psychology Department, Nottingham Trent University, Burton Street, Nottingham, NG1 4BU UK; 3 Department of Psychology, Faculty of Human Science, Arak University, Arak, 38156-8-8349 Iran

**Keywords:** Depression, Depression recovery, Cognitive reactivity, Psychometric validation, Leiden Index of Depression Sensitivity Revised, LEIDS-R

## Abstract

Cognitive reactivity (CR) to the experimental induction of sad mood has been found to predict relapse in recovered depressed patients. The Leiden Index of Depression Sensitivity Revised (LEIDS-R) is a self-report measure of CR. The aim of the present study was to establish the validity and reliability of the Persian version of the LEIDS-R. The participants were recovered depressed and non-depressed Iranian individuals (*n* = 833). The analyses included content validation, factor analysis, construct validity, and reliability testing. Preliminary construct validation analysis confirmed that factor analysis was appropriate for the Persian version of the LEIDS-R. Factor analysis displayed similar factor loadings to the original English version. The total internal consistency of the translated version, which was assessed using Cronbach’s alpha coefficient, was equal to 0.90. The test-retest reliability of the total score was equal to that of the test-retest conducted after a two-week interval at 0.94. Content validity, face validity, and construct validity, as well as reliability analysis were all found to be satisfactory for the Persian version of the LEIDS-R. The Persian version of the LEIDS-R appears to be valid and reliable for use in future studies, and has properties comparable to the original version and to that obtained in previous studies.

Major depressive disorder (MDD) is one of the most common psychiatric disorders in the young adult and adult population worldwide, with nearly 1 in 5 individuals in the general population suffering a lifetime major depressive episode (MDE) (Judd et al. 2000). Depressive disorders often begin at a young age, and can even occur in childhood (Kovacs 1992). Depressed individuals experience limitations in their usual day-to-day activities and are known to have higher health service utilization compared to non-depressed individuals (Johnson et al. 1992; Mazzucchelli et al. 2009; Mohr and Goodkin 1999). The World Health Organisation (WHO) indicated that over 350 million people worldwide suffer from depression disorder, with less than half of these individuals having access to treatment (WHO 2016). Among those that receive treatment, MDD is often a chronic, lifelong illness with a high risk for relapse and/or recurrence (Witkiewitz and Marlatt 2011). However, the course is highly variable among individuals. In one general population study, the average duration of a MDE was three months (Spijker et al. 2002).

Relatively little research into MDD has been carried out in Iran. A systematic review by Sadeghirad et al. (2010) reported 44 studies had reported the prevalence of MDD in Iranian populations (24 reporting the current prevalence and 20 reporting the lifetime prevalence). Based on these studies, the authors reported a current prevalence rate of 4.1 % of MDD among Iranian adults with women being almost two times more likely to suffer from it. There was no difference in prevalence rates of MDD between those living in urban or rural areas. The current (12-month) prevalence rate in Iran was reported as being lower than countries such as the USA (5–10 %), Ukraine (8.3 %), and New Zealand (5.7 %) but higher than countries such as Mexico (3.9 %), Spain (3.9 %), Japan (2.9 %), China (2 %), and Nigeria (1 %) (Sadeghirad et al. 2010). A systematic review of 53 Iranian adolescent studies found much higher prevalence rates of depression than in adults (13 % to 43.5 %) but the large majority of the studies were on small self-selected samples using a wide variety of different instruments (Sajjadi et al. 2013).

The apparently lower rate of adult MDD seen in Iran than some countries may be due to lack of ability to detect somatically-oriented depression. It may also be that depressive episode may be inherently somatic in manifestation in non-Western nations or ethnic groups. Thus, the current depression criteria, which are primarily psychologically based, might be insufficient to accurately assess depressive syndromes in countries like Iran, which may tend to normalize depression (Mohammadi et al. 2005; Mohammadi et al. 2006).

In secondary care settings, relapse and recurrence following recovery from MDD are common (Segal et al. 2006). The risk factors for depressive relapse/recurrence are multifaceted and involve a complex and dynamic interaction of biological, social, and psychological factors (Segal and Dobson 1992). The risk factors underlying relapse/recurrences may differ from risk factors that underlie the first onset of this disorder (Lewinsohn et al. 1999). One of these risk factors is cognitive reactivity (CR), that is based on Teasdale’s (1988) differential activation hypothesis. This hypothesis states that during a depressive episode, an association between dysphoric mood and negative thinking patterns is formed, such that the subsequent low mood will reactivate the negative thinking patterns (Alloy 2001; Beevers et al. 2011; Ginting et al. 2013; Ingram and Ritter 2000; Scher et al. 2005; Taylor and Ingram 1999). The reactivation of such dormant cognitive styles poses the risk of the individual suffering from another full episode of depression. CR is defined as the ease with which such maladaptive cognitions are triggered by non-pathological low mood (Lau et al. 2004; Van der Does 2002b). CR can be assessed in two ways: (a) by assessing cognition during a neutral mood and during a (naturally occuring or induced) sad mood state or (b) by self-report. In a mood induction procedure, negative thinking is assessed before and after the induction and the change score is the CR. In these experiments, recovered depressed typically have higher CR scores than never-depressed individuals (Lau et al. 2004; Van der Does 2002a), although some studies have found no differences (Van der Does 2005).

The Leiden Index of Depression Sensitivity Revised (LEIDS-R) is a self-report inventory of CR and was originally created in the Dutch language. LEIDS-R is useful for exploring a person’s history of past depression symptoms and suicidal tendencies when the person is in full remission, as well as determining behavioral reactivity to a mood challenge (Williams et al. 2008). Since 2003, an increasing number of researchers have studied the LEIDS-R with different subpopulations. These studies were mostly carried out in European countries but not in Iran. Due to its demonstrated effectiveness, there is a need to introduce the LEIDS-R inventory to the Persian culture to enable psychologists, counselors and clinicians to use in clinical settings.

Using the LEIDS-R, respondents indicate how their typical behaviors and cognition changes when they experience a low mood (e.g., “When in a low mood, I am more inclined to avoid difficulties or conflicts” and “When I feel sad, I feel more that people would be better off if I were dead”). Studies have shown that previously depressed individuals report significantly higher scores on LEIDS-R than non-depressed (ND) individuals (Booij and Van der Does 2007; Moulds et al. 2008; Kruijt et al. 2013). To date, translated versions of LEIDS-R in several other languages are in the process of being validated and developed. For instance, the LEIDS-R has been translated into Slovenian, Italian, German, French, Spanish, Farsi, Chinese and Arabic.

Currently, no valid or reliable self-report instrument to assess CR in depression or published reports on the acceptability and psychometric properties of the LEIDS-R in the Persian language exist. Considering that Persian is the first language of more than 110 million people in Iran, Pakistan, Tajikistan, and Afghanistan (Encyclopedia Britannica 2011), adapting and validating a Persian version of LEIDS-R is a timely and necessary step to progress research in a Persian context. Consequently, the main purpose of the present study was to adapt and validate the Persian version of LEIDS-R inventory to assess CR in the Iranian context.

## Materials and Methods

The present study was conducted in two stages, with first stage involving the translation and content validity process and the main study involving the administration of the test.

## Translation

The present study followed the rigorous steps in international translation, and are based on the guidelines from Beaton et al. (2000). In the LEIDS-R translation process, three psychologists from Iran with good command of the English and Persian languages performed forward translation independently. Initially, the translators disagreed on a few specific simple words in the inventory because they could be translated into closely related words. However, the original scales remained generally unchanged. Finally, after a professional iterative discussion, the translators agreed on the final wording of the translated scale.

During the second step, the Persian version was back translated by three professional translators who had not seen the original English version. After completing the back translation, they checked for correspondence between the two versions and to ensure adherence to the Iranian cultural context. An iterative discussion session was conducted that enabled the translators to reach an agreement on the most appropriate translation. At this stage, consensus in terms of grammar, idiomatic, semantic, and conceptual equivalence was reached. After considering suggestions from the judges, the pre-final version of the translated inventory was reviewed and approved with consensus from the committee, which included a panel of clinical psychologists and psychiatrists. The final step was the pre-test of the pre-final LEIDS-R (face validity). The pre-test was conducted with 20 individuals (10 never depressed individuals (ND) and 10 individuals recovered from depression (RD)). Each individual who completed the inventory was asked if any terms and sentences were unclear or difficult to understand. The pre-testing demonstrated that the instrument was very easy to complete for all 20 participants. Thus, the final version of the Persian version LEIDS-R was created and its reliability and validity were tested. Overall, the process of translation was straightforward and non-problematic. A copy of the Persian version is available from the authors.

## Content Validity

The content validity (CV) of an instrument can be determined by using the viewpoints of a panel of experts, who decide whether the items adequately represent the behaviors being assessed (Fitzner 2007). Qualitative testing was conducted by content and lay experts. Lay experts are the potential research participants, whereas content experts are professionals with experience in the field (Zamanzadeh et al. 2015). Using participants of the target group as experts ensures that the population for whom the instrument is being developed is represented (Zamanzadeh et al. 2015). Quantitative psychometric properties of Content Validity Ratio (CVR), Content Validity Index (CVI), and face validity were assessed using an expert panel and lay experts by assuming that the content of the program would be used as an educational assessment. The expert panel comprised 10 specialists in cognitive psychology, development psychology, and psychometrics. In calculating CVR, the expert panel was asked to evaluate each item using a three-point Likert scale: 1 = essential, 2 = useful but not essential, and 3 = unessential. Then, according to Lawshe’s table (Wynd et al. 2003), items with CVR scores of 0.56 or above were selected (Hajizadeh and Asghari 2011). For CVI, Waltz and Bausell’s (1981) recommendation was followed and the same panel were asked to evaluate the items according to a four-point Likert scale on ‘relevance’, ‘clarity’, and ‘simplicity’. A CVI score of ≥0.80 was considered acceptable (Naderimagham et al. 2012; Polit and Beck 2006).

## Face Validity

Face validity refers to an assessment of how lay individuals comprehend an instrument (Sarmugam et al. 2014). Qualitative and quantitative methods were applied to evaluate face validity. For the quantitative method, 20 individuals (10 RD and 10 ND) were asked to evaluate the instrument and score each item on a five-point Likert scale to evaluate ‘Item Impact Score’ (Impact Score = Frequency × Importance). An impact score of ≥1.5, which corresponds to a mean frequency of 50 % and a mean importance of three on the five-point Likert scale, was shown to be satisfactory as recommended (Sato and Ikeda 2015). In the qualitative phase, the same individuals were asked on the ‘relevance’, ‘ambiguity’, and ‘difficulty’ of the items. After determining face validity, some minor changes were made to the preliminary LEIDS-R questionnaire.

## Main Study

### Participants and Procedure

The present study comprised 833 participants, who were divided into RD (*n* = 391) and ND (*n* = 449), presenting a large enough sample for reliable output. Originally, 840 participants took part in the study (428 females and 405 males). However, seven individuals (1 %) reported to suffer currently from clinically significant levels of depression and were therefore excluded. Of the remaining sample (*N* = 833), 384 participants (48.61 %) were RD (M_age_ = 26.31 years; range = 18–56 years) and 449 participants (46 %) were ND (M_age_ = 24.60 years; range = 18–49 years). Their level of education varied from below diploma to PhD (see Table 1). Participants had to be at least 18 years of age and be fluent in Persian.

**Table 1:** Characteristics of study participants (*N* = 833)

	Recovered from Depression	Never Depressed
	(*n* = 384)	(*n* = 449)
Gender (% female)	57.3	69.5
Age in years (SD)	26.31 (5.34)	24.60 (5.81)
Marital Status (% single)	68.4	70.6
Educational level (% completed school)	90.3	94.7
Number of Depressive Episodes	2.37	___
Ethnicity (% of Sample)		
Fars	73.0	75.0
Other (Kord, Lor,Turk, and Arab)	35.0	20.0
Current Psychological Treatment (%)	21.8	___
Current Antidepressant Treatment(%)	18.4	-----
LEIDS-R Mean Score	91.21(10.33)	70.49(12.50)
Dysfunctional Cognitions Scale (DAS)	105.6(14.4)	101.7(16.9)

**Fig. 1:**
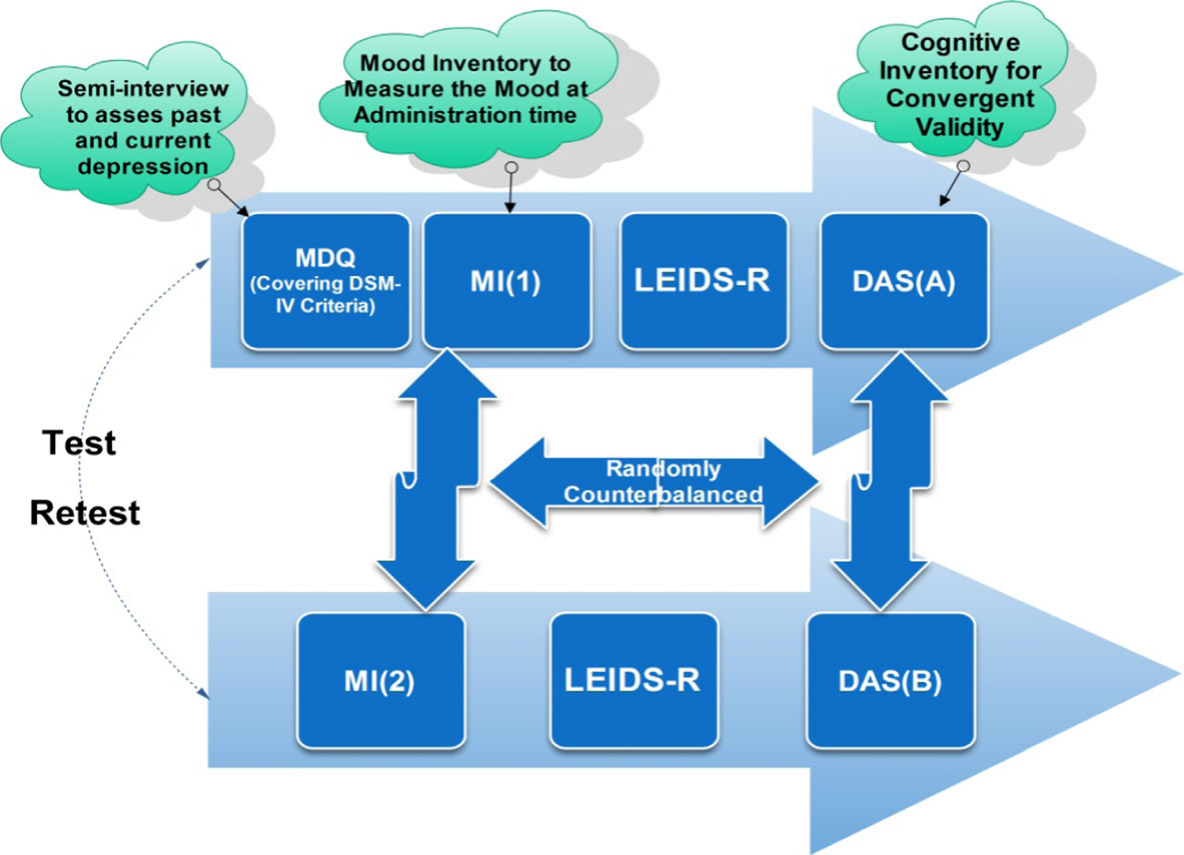
Overview of the test administration

The present study also involved a random selection of centers from different Cognitive Behavior Therapy (CBT) and Depression Center Consultation Clinic (DCC) centers in Iran. For the RD group, 384 individuals were contacted who had experienced at least one MDE during the past a year and had recovered completely. The RD respondents were contacted through e-mail or telephone, through which an appointment was made for them to come into the center and complete the inventory. The ND respondents were informed of the study through poster advertisements in a number of centers, universities, and schools in Iran. The respondents were tested twice individually using the same instruments (test-retest) during a two-week period. Each test took approximately 35 min during the first administration and approximately 15 min during the second administration. The psychologists and clinician who assisted in the administration of the inventories first introduced themselves. They then asked the participants to answer the self-report inventories honestly. The participants answered 75 items during the first administration and 56 items during the second administration. The test and re-test used two different booklets to counterbalance and avoid fatigue effects. These booklets were distributed randomly among the participants. During the first appointment, some of the participants were asked to complete the Major Depression Questionnaire (MDQ) to assess their past and current depression (covering DSM-IV criteria). Mood Inventory One (MI1), LEIDS-R, and Dysfunctional Attitude Scale (DAS)-A were then administered. Meanwhile, some of the paprticipants were asked to fill in the MDQ, Mood Inventory Two (MI2), LEIDS-R, and DAS-B (see Fig. 1). For the test-retest investigation, the psychologists and clinician scheduled a retest approximately two weeks after the first appointment. The retest also used two different test booklets (i.e., MI1, LEIDS-R and DAS-A and MI2, LEIDS-R and DAS-B). The participants who completed the first booklet in the first test were given the second booklet in the retest (Fig. 1). For each subscale, the participants were not allowed to omit more than one item to be included in the subsequent analysis (Van der Does 2002a). All participants signed an informed consent form to ensure that they were willing to take part in the study. Participants were also informed that participation in the study was optional and that all personal information would be kept confidential.

## Measures

### Demographic Information

Demographic information included education, age, and gender, which was obtained in interviews.

## Cognitive Reactivity to Sad Mood

## Leiden Index of Depression Sensitivity-Revised

LEIDS-R (Van der Does and Williams 2003) is a self-report scale that provides clinicians and researchers with a time-efficient means by which to assess CR to sad mood. This scale has been found to be predictive of the development of depressive symptoms as well as vulnerability to depression (Alloy 2001; Van der Does 2005). Before answering the LEIDS-R, the participants have to imagine being in a sad mood and indicate the degree to which a number of statements describe typical cognitions and behaviors if they were to experience such a sad mood (Raes et al. 2009). The scores on this test have been found to assess depression prevalence in multiple longitudinal studies and to correlate with depression risk factors (Moulds et al. 2008), genetic markers of depression (Antypa and Van der Does 2010), and reaction to tryptophan depletion (Booij Van der Does 2007). LEIDS-R comprises 34 items and six-subscales (Van der Does 2002a, b). The subscales are Hopelessness/Suicidality (HOP; five items with a maximum score of 20), Acceptance/Coping (ACC; five items with a maximum score of 20), Aggression (AGG; six items with a maximum score of 24), Control/Perfectionism (CON; six items with a maximum score of 24), Risk Aversion (RAV; six items with a maximum score of 24), and Rumination (RUM; six items with a maximum score of 24). Each item is rated on a five-point Likert scale ranging from 0 = ‘not at all’ to 4 = very strongly, with higher scores indicating higher CR to sad mood. The LEIDS-R total score is derived by adding up the scores from each subscale, with total scores ranging from 0 to 136. Internal consistency is 0.89 for the LEIDS total score, and ranges between 0.62 (Acceptance/Coping) and 0.84 for the subscales (Hopelessness/Suicidality; Steenbergen et al. 2015).

### Major Depression Questionnaire, Persian Edition

The Major Depression Questionnaire (MDQ) is a self-report inventory used to assess the presence of past and current major depression (Van der Does et al. 2003). Williams et al. (2008) stated that the MDQ comprises questions covering the DSM-IV criteria for current and past major depression (American Psychiatric Association 1994), and includes questions on its effects on the functioning and exclusion criteria, such as bereavement. In the present study, consistency of diagnoses derived by the questionnaire with diagnoses based on interviews was assessed in a subsample of 39 individuals from the present sample. These individuals participated in a face-to-face interview using the Structured Clinical Interview for DSM-IV (SCID, First et al. 2002). The MDQ demonstrated good consistency with diagnoses based upon the SCID (Williams et al. 2008). In the present study, MDQ was used to distinguish between RD and ND groups. The MDQ has been validated in Iran and used in previous studies (Barekatain et al. 2008).

### Mood Measurement Inventories, Persian Edition

The Mood Measurement Inventories (MIs) are self-report inventories that assess moods at various stages of the study (Phillips et al. 2002). The MIs have been divided into two different inventories (Akbari and Hommel 2012), Mood Inventory One (MI1) and Mood Inventory Two (MI2). MI1 includes descriptions of happy–sad, active–exhausted, peaceful–anxious, carefree–serious, and energetic–somber, whereas MI2 uses five adjectives with similar meaning, including positive–negative, lively–tired, calm–uptight, bright–dispirited, and cheerful–low. “The positive mood indicators (three hedonic, one physical arousal, and one worry measure) are presented at the left-hand side of a page, with the negative words on the right, and a scale from 1–9 written in numerals in between each word pair. Participants are asked to rate their current mood state with the numerical scales. Scores are totaled across the five word pairs” (Phillips et al. 2002; p.13). In the present study, MIs were used to record moods at various stages of the study, with higher total scores indicating happier or more positive moods (Perham and Oaksford 2005).

### The Dysfunctional Attitude Scale, Persian Edition

The Dysfunctional Attitude Scale (DAS) is a cognitive instrument that has been used widely in cognitive therapy research (Weissman and Beck 1978). The DAS was originally developed as a 100-item scale, but Weissman (1979) developed it into two parallel 40-item scales (DAS-A and DAS-B). Subsequently, Power et al. (1994) developed the 24-item DAS (DAS-24) based on DAS-A and DAS-B. DAS was included in the present study to assess depressogenic schemata and the presence of dysfunctional attitudes that may relate cognitive vulnerability to depression. Each item is rated on a seven-point Likert scale ranging from 7 = “totally agree” to 1 = “totally disagree”. Higher total scores indicate that the participant’s attitude is more dysfunctional. The total DAS scores range from 40 to 280 and scores higher than 125 indicate dysfunctional attitudes. In the present study, DAS was used to determine concurrent validity. DAS has been validated in the Persian language and used previously in other studies (Talepasand et al. 2010).

### Iranian Validation of the LEIDS-R Scale

CVR and CVI were performed to assess content validity of the LEIDS-R. The present study also used exploratory factor analysis (EFA) and confirmatory factor analysis (CFA) to determine construct validity. EFA was conducted utilizing principal component analysis (PCA) with varimax rotation. The criterion for retaining the items was item loading ≥0.4. CFA was employed through structural equation modeling. The present study utilized six recommended indices (comparative fit index [CFI], Goodness-of-fit index [GFI], Adjusted goodness of fit index [AGFI], root mean square error of approximation [RMSEA], chi-square/df, and standardized root mean square residual [SRMR]; (Schermelleh-Engel et al. 2003). The growth curve model fitted if the CFI, GFI and AGFI were greater than 0.90. Relative chi-squares less than 5.00 and RMSEA and SRMR values less than 0.08 were considered acceptable (Schermelleh-Engel et al. 2003). The present study analyzed the data using SPSS version 21 (SPSS Inc., Chicago, IL, USA) for EFA and reliability. AMOS 21 was used for CFA. The internal consistency of the scale was evaluated by calculating Cronbach’s alpha (α) coefficient. The test-retest intraclass correlation coefficient was used in the reproducibility analysis of LEIDS-R.

## Results

**Fig. 2:**
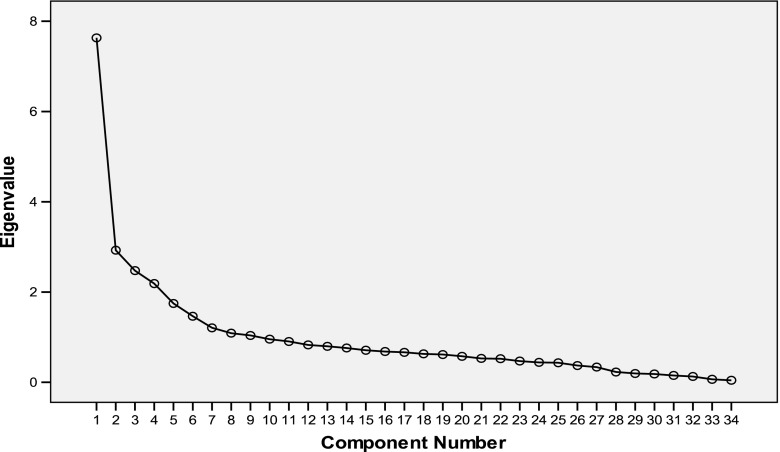
Scree plot for Persian version of LEIDS-R test

Psychometric analysis of the LEIDS-R demonstrated that the factor solution found was adequate. The result of the Kaiser-Meyer-Olkin test was 0.88 and Barlett sphericity was significant (Chi-Square = 9864.6; gl = 46; *p* = 0.001). Six “factors” were determined and accounted for about 54.20 % of the variance (see Table 2). The saturation of each item on the PCA is presented in Table 3. The sedimentation graph shows a suitable six-factor solution (see Fig. 2).

**Table 2:** Eigenvalues and Factor Loadings for LEIDS-R

Initial Eigenvalues				
Component	Total	% of Variance	Cumulative %	Total
1	7.631	22.444	22.444	3.615
2	2.926	8.607	31.051	3.286
3	2.474	7.276	38.327	4.161
4	2.187	6.433	44.760	3.371
5	1.748	5.140	49.900	3.353
6	1.465	4.308	54.208	2.439

**Table 3:** Factor Structure of the 34-item LEIDS-R

Items		saturation
1	I can only think positive when I am in a good mood.	0.812
2	When in a low mood, I take fewer risks.	0.841
3	When I feel sad, I spend more time thinking about what my moods reveal about me as a person.	0.815
4	When in a sad mood, I am more creative than usual.	0.833
5	When I feel down, I more often feel hopeless about everything.	0.841
6	When I feel down, I am more busy trying to keep images and thoughts at bay.	0.834
7	In a sad mood, I do more things that I will later regret.	0.858
8	When I feel sad, I go out and do more pleasurable activities.	0.811
9	When I feel sad, I feel as if I care less if I lived or died.	0.832
10	When I feel sad, I am more helpful.	0.869
11	When I feel sad, I am less inclined to express disagreement with someone else.	0.824
12	When I feel somewhat depressed, I think I can permit myself fewer mistakes.	0.812
13	When I feel down, I more often feel overwhelmed by things.	0.850
14	When in a low mood, I am more inclined to avoid difficulties or conflicts.	0.806
15	When I feel down, I have a better intuitive feeling for what people really mean.	0.812
16	When in a sad mood, I become more bothered by perfectionism.	0.841
17	When I feel sad, I more often think that I can make no one happy.	0.875
18	When I feel bad, I feel more like breaking things.	0.826
19	I work harder when I feel down.	0.864
20	When I feel sad, I feel less able to cope with everyday tasks and interests.	0.804
21	In a sad mood, I am bothered more by aggressive thoughts.	0.832
22	When I feel down, I more easily become cynical (blunt) or sarcastic.	0.817
23	When I feel down, I feel more like escaping everything.	0.852
24	When in a sad mood, I feel more like myself.	0.809
25	When I feel down, I more often neglect things.	0.801
26	When I feel sad, I do more risky things.	0.838
27	When I am sad, I have more problems concentrating.	0.822
28	When in a low mood, I am nicer than usual.	0.843
29	When I feel down, I lose my temper more easily.	0.862
30	When I feel sad, I feel more that people would be better off if I were dead.	0.827
31	When I feel down, I am more inclined to want to keep everything under control.	0.814
32	When I feel sad, I spend more time thinking about the possible causes of my moods.	0.832
33	When in a sad mood, I more often think about how my life could have been different.	0.805
34	When I feel sad, more thoughts of dying or harming myself go through my mind.	0.849

**Fig. 3:**
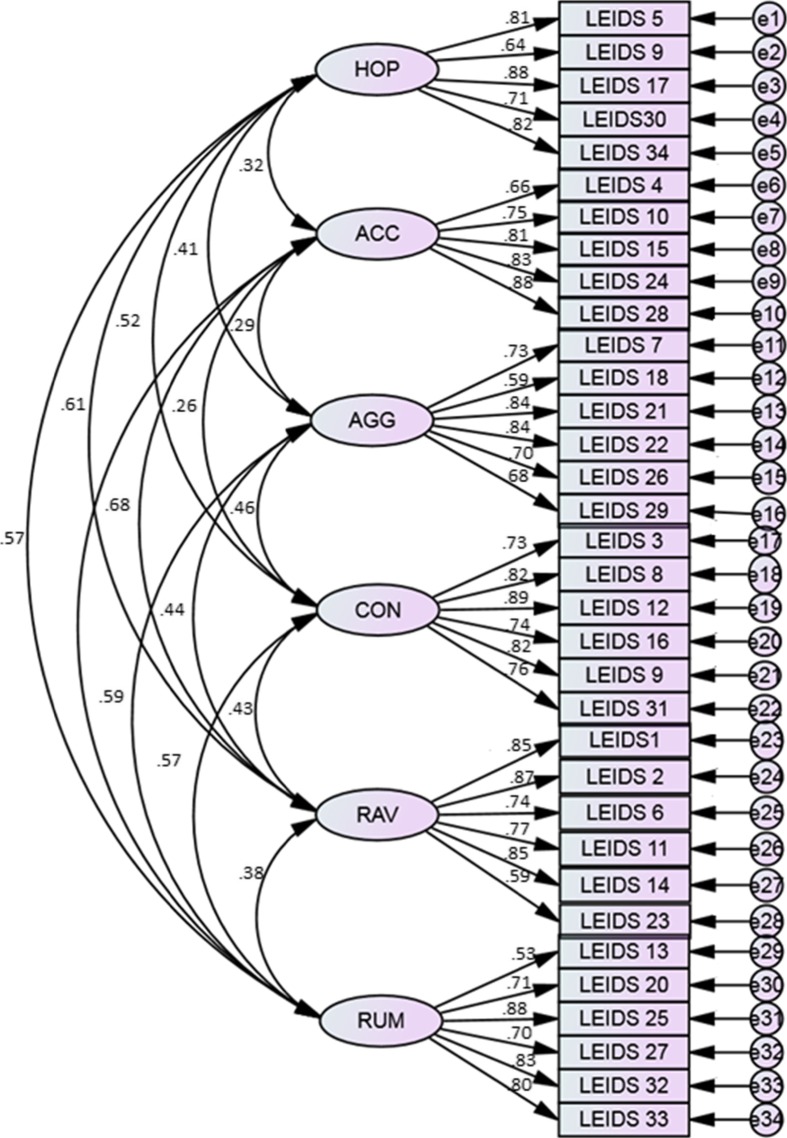
CFA of the Persian LEIDS-R version

Construct validity testing was carried out based on the construct reliability of the sub-structures, convergent validity, and discriminant validity (Kim and Kim 2010). The model fit test of the total structure for each item was determined through CFA. The model fit of LEIDS-R was analyzed using CFA. The goodness of fit index of the model that included all 34 items was χ^2^ = 254.27 (df 136), RMR = .04, GFI = .89, AGFI = .90, CFI = .90, TLI = .87, RMSEA = .050, and SRMR = .046. A comparison of the analysis with 31 items (eliminating the three items with low factor loadings) produced the following values: χ^2^ = 250.24 (df 134), RMR = .04, GFI = .90, AGFI = .92, CFI = .91, TLI = .89 RMSEA = .039, and SRMR = .031 (see Table 5 and Fig. 3). The difference in values compared with the goodness of fit index of the 34 items was very slight, and by excluding the three items, did not appear to enhance the adequacy of the entire model. Therefore, including all 34 items as the final model was more appropriate. One advantage of using the full-scale model is that international comparisons between countries are possible. Consequently, the Persian version of LEIDS-R can be used as an assessment instrument of CR in Iran.

Cronbach’s alpha coefficient was 0.90 for the 34 items of the Persian version of LEIDS-R and was within the accepted range of internal consistency. Table 4 demonstrates that the reliability for the different components in this scale ranged from 0.78 to 0.91. Test-retest reliability was examined in a subsample of 200 participants randomly selected from all sample participants who completed the questionnaire twice, once when they were convened to participate in the study and the second time two weeks later. Table 4 presents the inter-item correlation matrix between the test and retest items. With the exception of tiredness, fatigability, and sleepiness during the daytime, the correlation coefficients for the test and retest values of all items were ≥0.80, with a correlation coefficient for the total score of 0.94 (see Tables 4 and 5).

**Table 4:** Factor loadings of LEIDS-R questionnaire in six obtained factors

Subscale	Item							Test-Retest
		Component						
		1	2	3	4	5	6	
HOP	leids5	.661						0.83
	leids9	.709						0.91
	leids17	.573						0.86
	leids30	.810						0.84
	leids34	.636						0.90
ACC	leids4		.654					0.91
	leids10		.880					0.86
	leids15		.473					1.00
	leids24		.687					0.92
	leids28		.595					0.88
AGG	leids7			.789				0.84
	leids18			.585				0.96
	leids21			.714				0.81
	leids22			.386				0.87
	leids26			.627				0.90
	leids29			.530				0.93
CON	leids3				.707			0.80
	leids8				.648			0.96
	leids12				.391			0.87
	leids16				.718			0.92
	leids19				.629			0.82
	leids31				.702			1.00
RAV	leids1					.746		0.86
	leids2					.419		0.82
	leids6					.661		0.90
	leids11					.533		1.00
	leids14					.620		0.95
	leids23					.609		0.89
RUM	leids13						. 642	0.80
	leids20						.590	0.96
	leids25						.661	0.83
	leids27						.496	0.85
	leids32						.713	0.93
	leids33						.394	0.89
Eigen value		7.6	2.9	2.4	2.1	1.7	1.4	
Cronbach’s alpha,(Total α = 90)		.79	.81	.83	.91	.80	.78	0.94
Mean (SD) Total = 59.87(17.31)		9.05(3.42)	8.25(3.07)	10.35(3.95)	10.50(3.86)	10.74(3.89)	10.95(3.92)	

Extraction method: principal component analysis

Rotation Method: Oblimin with Kaiser Normalization

a. Rotation converged in 7 iterations

**Table 5:** Model Fit Indices for LEIDS-R from Confirmatory Factor Analyses

	*χ* ^2^(*p*)	df	Q	RMR	GFI	AGFI	CFI	TLI	RMSEA	SRMR
Model (34 items)	254.27	136	2.46	.04	.89	.90	.89	.88	.050	.046
Modification Model (32 items)	250.24	134	2.32	.04	.90	.91	.90	.90	.039	.031

Q: χ2/df; RMR: Root Mean Squared Residual; GFI: Goodness Fit Index; AGFI: Adjusted Goodness Fit Index; CFI: Comparative Fit Index; TLI: Tucker-Lewis Index

Concurrent validity exhibited good correlation between LEIDS-R and DAS inventories for RD and ND groups (see Table 6). The correlation between CR scores for LEIDS-R and DAS was significant, with a value of 0.67 (*p* < 0.01). No significant differences were found in LEIDS-R scores in relation to sociodemographic variables (sex, age, marital status, and education).

**Table 6:** Correlations Between LEIDS-R and DAS

Correlations				
			LEIDSR	DAS
RD	LEIDS-R	Pearson Correlation	1	.691^**^
		Sig. (2-tailed)		.000
ND		Pearson Correlation	1	. 602^**^
		Sig. (2-tailed)		.000

Correlation is significant at the 0.01 level (2-tailed)

## Discussion

At present, most studies on vulnerability to depression and CR in Iran have used scales (e.g., the DAS) that cannot distinguish between recovered and non-depressed individuals (Segal et al. 1999). To the best of our knowledge, the present study is the first examination of the construct of in an Iranian context using the Persian version of LEIDS-R. The present study was conducted to evaluate the reliability and validity of LEIDS-R that assesses CR. The present study, which simplified the Persian version of LEIDS-R, obtained impressive and robust results in relation to its psychometric properties. After the translation process to the Persian language -to provide a self-report questionnaire for the assessment of CR in the Persian language– this scale was administered to individuals who had recovered from depression. However, to prepare a psychometric instrument applicable to a target population, obtaining a desirable score for translation quality across aspects of clarity, common language, conceptual equivalence, and total quality of translation, is important. The fluency and understandability of LEIDS-R was confirmed by the participants.

Internal consistency is a type of reliability that shows how every component of the scale has the ability to assess good variance. In the present study, item-total correlation analysis was used to demonstrate that internal consistency had statistically significant values to the total score (*p* < 0.05). This result suggests that the results are consistent with the original study. Cronbach’s alpha coefficients were good for all scales, indicating adequate internal reliability. Stability of the scale was also exceptionally robust, with demonstrably high test–retest reliability. Test-retest reliability was confirmed by ICC for the subscales, and ICC test-retest ranged from 0.64 to 0.89. These results are consistent with those obtained in other studies (Solis 2015; Van der Does 2005; Van der Does 2002a). The cumulative contributions of these factors to the total variance were 54.2 % (reference, >0.4). All items had relatively high loadings on the corresponding common factors and low loadings on other factors, indicating that the Persian version of LEIDS-R had good construct validity (Beavers et al. 2013). The results observed using CFA were largely consistent with those reported in previous studies (Solis 2015). Furthermore, the six-factor model displayed adequate fit and all items loaded strongly onto the expected latent factor, and the factor structure was maintained cross-culturally.

The present study provides valuable information relating to the Persian version of the LEIDS-R, which could be useful in interpreting scores of this instrument. However, the results of the present study were only a first approximation of the LEIDS-R psychometric properties among a Persian population. Hence, to accumulate further evidence of the validity of the scores on LEIDS-R, future studies should examine its factorial invariance using multigroup CFA (Weston and Gore 2006) and other recent methodological strategies, such as the exploratory structural equation modeling, which is an integration of EFA and CFA (Marsh et al. 2009). These analyses could contribute to ascertaining that metric relationships among the manifest variables and factors to examine if they behave in a similar fashion for different groups (i.e., gender and ethnic groups). As expected, there was convergent validity between the Persian version of LEIDS-R and the Persian version of DAS as demonstrated by the high Pearson correlation. The present study is not without its limitations. Because of the lack of similar reports concerning the reliability and validity in other language versions of LEIDS-R with which the present study could compare its results and cross-sectional design, as well as the lack of retrospective assessment of previous depressive symptoms, the present investigation is only the first step towards the introduction of a Persian version of LEIDS-R. The present study only examined content validity, construct validity, and reliability but other types of psychometric evaluation of the Persian LEIDS-R could be tested in future studies (e.g., invariance, and criterion and discriminant validity). Another limitation is that the results of the present study cannot be generalized to samples outside of Iran. Further studies should be conducted involving different sub-populations in Iran, such as depression and/or rumination, and which are representative of the Iranian patient group, using the LEIDS-R to asseses the utility of CR theory. Adapting and validating other instruments along with LEIDS-R, which are related to CR theory, such as The Beck Depression Inventory (BDI) (Beck et al. 1996) or the Hospital Anxiety and Depression Scale (HADS) (Zigmond and Snaith 1983), would be a valuable step. Ultimately the adapted and validated Persian version of LEIDS-R appears to be a good instrument to use in Iranian health centers to educate practitioners about CR and enable its use in the effective treatment of patients.

In conclusion, the present study reports the successful translation of LEIDS-R into Persian according to internationally recognised methodological standards. The Persian version of LEIDS-R has excellent internal consistency and high test-retest reliability as well as concurrent validity, as shown by the significant correlation with other scales reflecting depression. A six-factor structure, comparable to the original version, suggests adequate factorial validity of the Persian version of LEIDS-R. The Persian version of LEIDS-R is a robust psychometric measure that can be used for screening and epidemiological studies, and will be useful for assessing cognitive reactivity among the Persian-speaking population.
